# The effect of swimming exercise and diet on the hypothalamic inflammation of ApoE-/- mice based on SIRT1-NF-κB-GnRH expression

**DOI:** 10.18632/aging.103323

**Published:** 2020-06-09

**Authors:** Xialei Wang, Jingda Yang, Taotao Lu, Zengtu Zhan, Wei Wei, Xinru Lyu, Yijing Jiang, Xiehua Xue

**Affiliations:** 1 The Affiliated Rehabilitation Hospital, Fujian University of Traditional Chinese Medicine, Fuzhou 350003, China; 2 College of Rehabilitation Medicine, Fujian University of Traditional Chinese Medicine, Fuzhou 350112, China

**Keywords:** hypothalamic inflammation, swimming exercise, diet control, SIRT1, GnRH

## Abstract

A high-fat diet and sedentary lifestyle could accelerate aging and hypothalamic inflammation. In order to explore the regulatory mechanisms of lifestyle in the hypothalamus, swimming exercise and diet control were applied in the high-fat diet ApoE-/- mice in our study. 20-week-old ApoE-/- mice fed with 12-week high-fat diet were treated by high-fat diet, diet control and swimming exercise. The results showed that hypothalamic inflammation, glial cells activation and cognition decline were induced by high-fat diet. Compared with the diet control, hypothalamic inflammation, glial cells activation and learning and memory impairment were effectively alleviated by swimming exercise plus diet control, which was related to the increasing expression of SIRT1, inhibiting the expression of NF-κB and raising secretion of GnRH in the hypothalamus. These findings supported the hypothesis that hypothalamic inflammation was susceptible to exercise and diet, which was strongly associated with SIRT1-NF-κB-GnRH expression in the hypothalamus.

## INTRODUCTION

The noticeable feature of aging is the degradation of various physiological functions with increasing age and eventually leading to lifespan. Aging is not just determined by a single but a variety of factors, including the neuroendocrine. Factors affecting the synthesis, secretion, and regulation of the neuroendocrine system contribute to aging. Recent studies have confirmed that the hypothalamus, as a neuroendocrine and autonomous regulatory center, plays a fundamental role in aging development and lifespan control. Hypothalamic inflammation is considered to be the common basis of metabolic syndrome and aging.

Diet and exercise are the key factor of aging. High-energy diets and sedentary lifestyles promote aging [1]. Studies have been showing that intake of a high-energy diet would induce metabolic disturbances and inflammation in the hypothalamus [2]. The hypothalamic inflammation is manifested by the activation of inflammatory pathways and glial cells, which are related to aging process [3]. The activation of NF-κB and inflammation in hypothalamus increased along with aging. Inhibition of NF-κB activation promoted the release of gonadotropin-releasing hormone (GnRH) in the hypothalamus, which played a crucial role in aging. Treatment with GnRH for 5-8 weeks accelerated neurogenesis and mitigated aging [4].

Aerobic exercise is a healthy lifestyle for regulating metabolism and inflammation, reducing the risk of chronic diseases and aging-related diseases [5, 6]. Studies have proved that aerobic exercise is essential for neural regeneration, apoptosis, inflammation and energy metabolism in the hypothalamus [7–9]. It is reported that aerobic exercise alleviates hypothalamus inflammation and has an anti-aging effect, of which the mechanism has not yet been clarified [10].

Silent Information Regulator 1 (SIRT1) is a longevity gene that linked to the extension of lifespan in many organisms. SIRT1 has various physiological functions by regulating some key targets via deacetylation, such as NF-κB and peroxisome proliferator-activated receptor γ (PPARγ) [11]. Chen WK et al. [12] demonstrated that long-term aerobic exercise facilitated SIRT1 protein expression and enhanced SIRT1-related anti-aging signals. Aerobic exercise induced the expression of SIRT1 which inhibited the NF-κB pathway. SIRT1-NF-κB was involved in the process of aging and neurodegenerative diseases [11, 13]. Swimming is one of the ideal ways for aerobic exercise [9, 14, 15]. It is estimated that swimming exerts neuroprotection and alleviates hypothalamic inflammation [9]. We therefore hypothesized that swimming exercise may have the effect of anti-aging by inhibiting the inflammation of the hypothalamus, which was associated with the regulation of SIRT1-NF-κB pathway and expression of GnRH. This study aimed to investigate the effects of swimming exercise on the peripheral and central inflammatory response and to explore the relationship between SIRT1-NF-κB-GnRH in the hypothalamus and aging.

## RESULTS

### Swimming exercise and diet control improved physiological conditions

**Figure 1:**
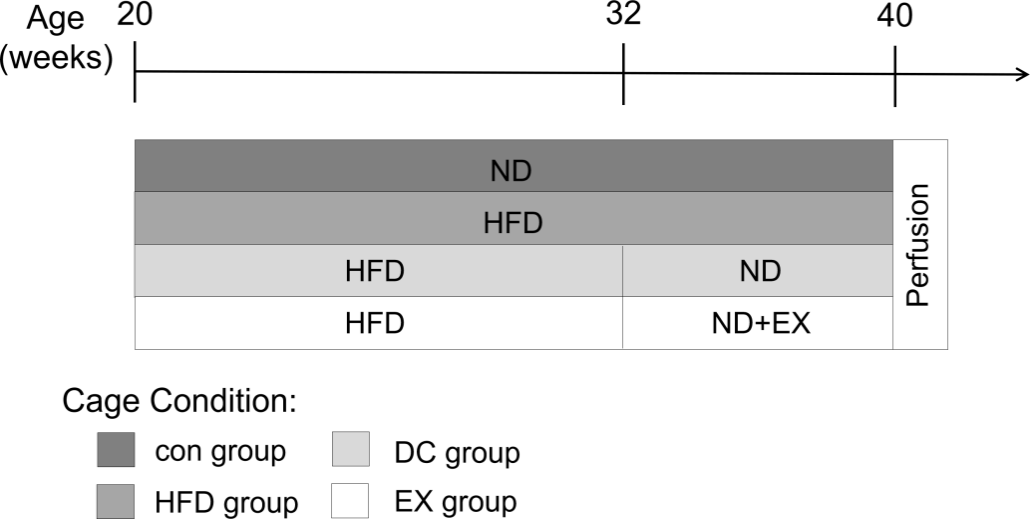
**Experimental design.** ApoE-/- mice were fed with a high-fat diet (HFD) at 20 weeks of age for 12 weeks, and then randomly divided into HFD group, DC group and EX group. The HFD group continued to have a high-fat diet, the DC group changed to a normal diet(ND), and the EX group performed an eight-week swimming exercise based on the normal diet. C57BL/6J mice with the same genetic background at the age of 20 weeks were used as a control group, and they continued to be fed with a normal diet throughout the experiment. At the end of the protocol, mice were deeply anesthetized with isoflurane and then decapitated.

**Figure 2:**
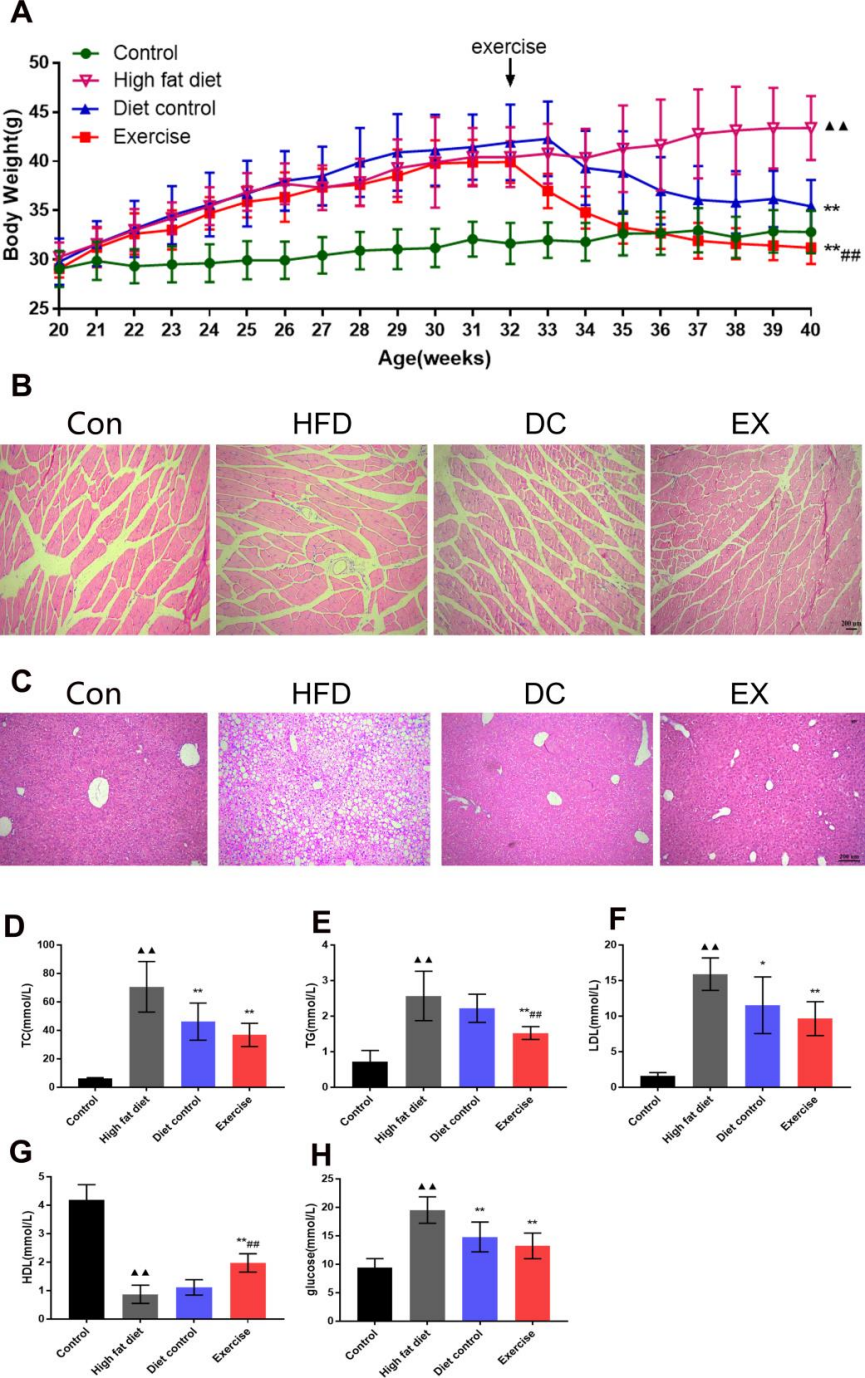
**Swimming exercise and diet control improved physiological conditions.** (**A**) The changes in body weight of mice at 20-40 weeks of age, respectively. There was no difference in body weight among groups at 32 weeks of age. It was significantly different in the body weight at the end of the intervention (40 weeks) among groups, EX group had the lowest body weight (*p* < 0.01); (**B**) HE staining of mouse gastrocnemius muscle. Scale bar = 200μm. (**C**) HE staining of mouse liver tissue. Scale bar = 200μm. (**D**–**H**) Serum TC, TG, LDL, HDL and glucose levels. vs control group, ^**▲▲**^*p* < 0.01. vs HFD group, *p < 0.05, ** *p* < 0.01. vs DC group, ^#^*p* < 0.05, ^##^ *p* < 0.01.

Mice were fed with a high-fat diet at 20 weeks of age, and then started swimming exercises and/or diet control interventions at 32 weeks of age. The specific experimental protocol is illustrated in Figure 1. The body weight of the mice was recorded weekly throughout the experiment. ApoE-/- mice were fed with a high-fat diet for 12 weeks. There was no significant difference in body weight among the high-fat diet (HFD) group, diet control (DC) group, and swimming exercises (EX) group. HFD group were fed with a high-fat diet continually and gradually gained weight with age. After diet control and swimming training for 8 weeks, both DC and EX group gradually lost the body weight compared to the HFD group (35.42 ± 2.70, 31.22 ± 1.64 vs. 43.38 ± 3.25; *p* < 0.01) (Figure 2A). The EX group had the lowest body weight among three groups (31.22 ± 1.64; *p* < 0.01) (Figure 2A). Gastrocnemius HE staining showed that high-fat diet induced inflammatory cell infiltration into muscle and muscle fibers irregularly distributed, which were improved by swimming exercise and diet control (Figure 2B). Same results were seen in the liver HE staining. Swimming exercise and diet control relieved the lipids deposit and inflammatory cells infiltration of the liver, reversed the degeneration of hepatocyte vacuoles, improved the integrity of liver lobular structure after high-fat diet feeding (Figure 2C). It indicates that swimming exercise and diet control are effective ways to maintain physiological functions.

Assay kits were used to evaluate the levels of TC, TG, LDL, HDL and glucose. The results showed that levels of TC, TG, LDL and glucose in the HFD group were significantly higher than those in the control group (*p* < 0.01) (Figure 2D–2F, 2H), and HDL levels in the HFD group were significantly lower than those in the control group (0.87 ± 0.32 vs. 4.18 ± 0.53; *p* < 0.01) (Figure 2G). Compared with the HFD group, the TC, LDL and glucose levels in the DC group were significantly reduced (TC: 70.54 ± 17.76 vs. 46.09 ± 13.04; *p* < 0.01. LDL: 15.89 ± 2.28 vs. 11.53 ± 3.98; *p* <0.05. glucose: 19.52 ± 2.32 vs.14.79 ± 2.61; *p* < 0.01) (Figure 2D, 2F, 2H), the TC, TG, LDL and glucose levels in the EX group were dramatically decreased (TC: 36.79 ± 8.14; TG: 1.52 ± 0.18; LDL: 9.64 ± 2.38; glucose: 13.22 ± 2.25; *p* < 0.01) (Figure 2D, 2E, 2H), and the HDL levels were significantly increased (1.97 ± 0.32; *p* < 0.01) (Figure 2G). There was significant difference of TG and HDL levels between the DC group and EX group (TG: 2.22 ± 0.40 vs. 1.52 ± 0.18; *p* < 0.05. HDL: 1.11 ± 0.27 vs. 1.97 ± 0.32; *p* < 0.01) (Figure 2E, 2G). It suggests that diet control and swimming exercise can suppress the serum levels of TC, TG, LDL and glucose, swimming exercise adds further benefits on top of those provided by the diet control.

### Serum SIRT1, IL-1β, TNFα, MMP-2, MMP-9 levels in the ApoE-/- mice

**Figure 3:**
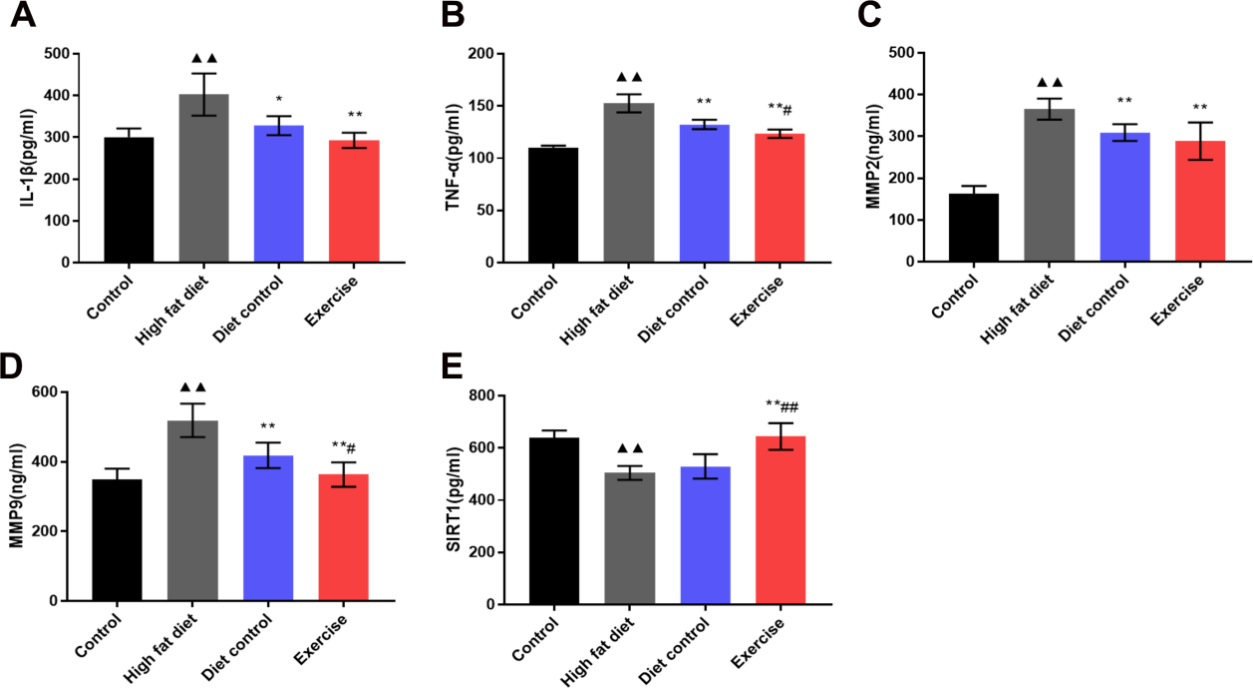
**Serum IL-1β, TNFα, MMP-2, MMP-9 and SIRT1 expression.** (**A**) The levels of IL-1β in serum. (**B**) The levels of TNFα in serum. (**C**) The levels of MMP-2 in serum. (**D**) The levels of MMP-9 in serum. vs control group, (**E**) The levels of SIRT1 in serum. ^**▲▲**^*p* < 0.01. vs HFD group, *p < 0.05, **p < 0.01. vs DC group, ^#^*p* < 0.05.

We hypothesized that swimming alleviated the inflammatory mediators in peripheral blood through SIRT1 regulation. High-fat diet induced the over-expression of IL-1β, TNFα, MMP-2, and MMP-9 and significantly inhibited the SIRT1 expression as opposed to the control group (*p* < 0.01. Figure 3). Compared with the HFD group, the levels of TNFα, MMP-2, and MMP-9 decreased in the DC group (TNFα:132.42 ± 4.44 vs. 152.62 ± 8.69; MMP-2: 309.38 ± 19.90 vs. 365.43 ± 25.29; MMP-9:418.10 ± 36.47 vs. 518.59 ± 48.01; *p* < 0.01) (Figure 3B–3D), the levels of IL-1β, TNFα, MMP-2 and MMP-9 in the EX group decreased (IL-1β: 292.91 ± 18.15; TNFα:123.38 ± 4.03; MMP-2: 288.89 ± 44.77; MMP-9: 363.19 ± 35.05; *p* < 0.01) (Figure 3A–3D), and SIRT1 levels in the EX group increased (644.20 ± 50.89; *p* < 0.01) (Figure 3E). Compared with the DC group, TNFα and MMP-9 decreased in the EX group (TNFα: 123.3 ± 4.03 vs. 132.42 ± 4.44; *p*<0.05. MMP-9: 363.19 ± 35.05 vs. 418.10 ± 36.47; *p* < 0.05) (Figure 3B, 3D), and SIRT1 increased significantly (644.20 ± 50.89 vs. 529.68 ± 46.74; *p* < 0.01) (Figure 3E). The data showed that diet control plus swimming exercise could better elevate SIRT1 expression and reduce the levels of TNFα and MMP-9 than diet control alone.

### Immunoblotting to detect hypothalamic protein expression

**Figure 4:**
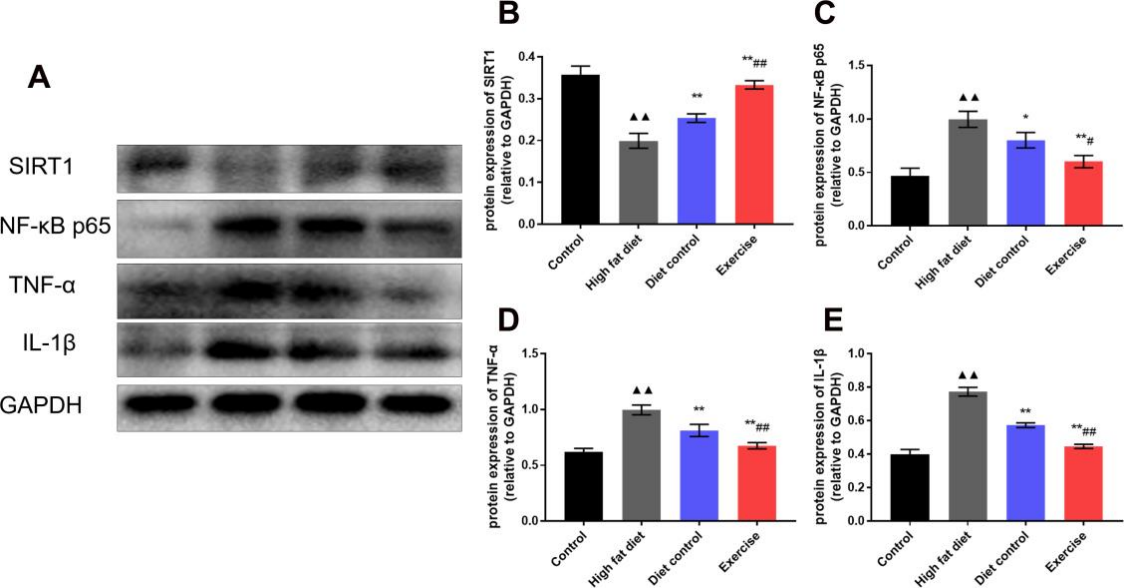
**The expression of SIRT1, NF-κB, TNFα, and IL-1β proteins in the hypothalamus.** (**A**) Western blot to detect the expression of SIRT1, NF-κB p65, TNF-α and IL-1β of the hypothalamus. (**B**) The expression of SIRT1 in the hypothalamus. (**C**) The expression of NF-κB p65 in the hypothalamus. (**D**) The expression of TNF-α in the hypothalamus. (**E**) The expression of IL-1β in the hypothalamus. vs control group, ^▲▲^*p* < 0.01. vs HFD group, *p < 0.05, ** *p* < 0.01. vs DC group, ^#^*p* < 0.05, ^##^ *p* < 0.01.

It is demonstrated that exercise and diet are the main factors of hypothalamic inflammation during aging. SIRT1 is a longevity gene and has the function of lifespan extension. It is believed that SIRT1 exerts a crucial role in aging-related hypothalamic dysfunction. To explore the effects of diet and swimming exercise on hypothalamic inflammation related to SIRT1 mediated pathways, western blot was used to detect the expression of SIRT1, NF-κB, TNFα, and IL-1β proteins in the hypothalamus (Figure 4A). The results showed that HFD group had the lower SIRT1 expression than control group (0.19 ± 0.02 vs. 0.36 ± 0.02; *p* <0.01) (Figure 4B), the levels of SIRT1 expression in DC and EX group increased gradually (0.25 ± 0.01, *p* <0.01; 0.33 ± 0.01, *p* <0.01) (Figure 4B). The expression of NF-κB raised dramatically in HFD group (1.00 ± 0.08 vs. 0.47 ± 0.07; *p* <0.01), DC group and EX group significantly inhibited the expression of NF-κB (0.80 ± 0.07, *p* <0.01; 0.60 ± 0.06, *p* <0.01) (Figure 4C), the NF-κB expression in EX group was lower than that in DC group (0.60 ± 0.06 vs. 0.80 ± 0.07, *p* <0.05) (Figure 4C). The same tendency was observed in the expression of TNFα and IL-1β (*p* < 0.05)(Figure 4D, 4E). Compared with the DC group, EX group had the higher SIRT1 expression (*p* <0.01) (Figure 4B) and the lower NF-κB, TNFα and IL-1β expression (*p* < 0.05, *p* < 0.01 and *p* < 0.01) (Figure 4C–4E). It indicates that the NF-κB expression may be mediated by SIRT1 and affect inflammation of the hypothalamus. The data is consistent with the benefits of diet and swimming exercise on the hypothalamic inflammation.

### Immunohistochemical assay for activation of microglia and astrocytes in the hypothalamus

**Figure 5:**
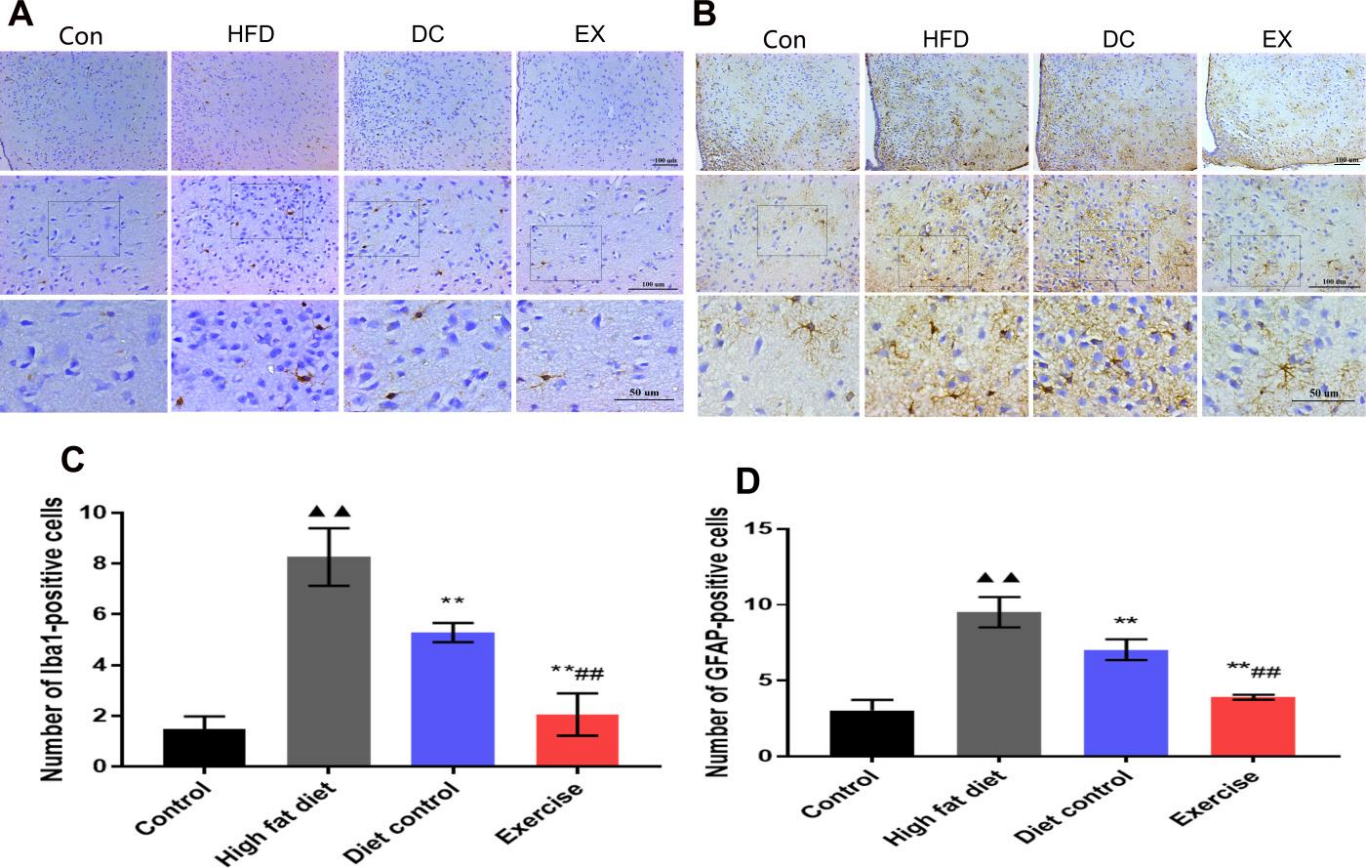
**The activation of microglia and astrocytes in the hypothalamus.** (**A**) Iba1 expression levels in hypothalamic microglia. The statistics analysis of the expression of Iba1 positive cells. (**B**) The expression levels of GFAP in hypothalamic astrocytes. (**C**) The statistics analysis of the expression of Iba1 positive cells. (**D**) The statistics analysis of the expression of GFAP positive cells. The scale bar is shown in the figure. vs control group, ^▲▲^*p* < 0.01. vs HFD group, ** *p* < 0.01. vs DC group, ^##^ *p* < 0.01.

Since microglial activation can promote inflammation and activate A1-reactive astrocytes. It is believed that the activation of microglia and astrocytes is involved in the hypothalamic inflammation and aging process. Immunohistochemical assay was applied to analyze the expression of Iba1 and GFAP in the hypothalamus (Figure 5A, 5B). The expression of Iba1 and GFAP in the hypothalamus was higher in the HFD group than the control group (Iba1: 8.26 ± 1.13 vs. 1.48 ± 0.50, *p* < 0.01; GFAP: 9.50 ± 1.00 vs. 3.05 ± 0.68, *p* < 0.01)(Figure 5C, 5D), and the expression of Iba1 and GFAP in the DC group were lower than the HFD group (Iba1: 5.29 ± 0.38 vs. 8.26 ± 1.13, *p*<0.01; GFAP: 7.03 ± 0.68 vs. 9.50 ± 1.00, *p* < 0.01)(Figure 5C, 5D). The expressions of Iba1 and GFAP in the EX group were lower than the DC group (Iba1: 2.05 ± 0.83 vs. 5.29 ± 0.38, *p* < 0.01. GFAP: 3.90 ± 0.16 vs. 7.03 ± 0.68, *p* < 0.01) (Figure 5C, 5D). It suggests that microglia and astrocytes act synergistically in the hypothalamic inflammatory response. Swimming exercise and diet control can effectively suppress the activation of glial cells in the hypothalamic inflammation.

### Immunohistochemical assay for GnRH expression in hypothalamus

**Figure 6:**
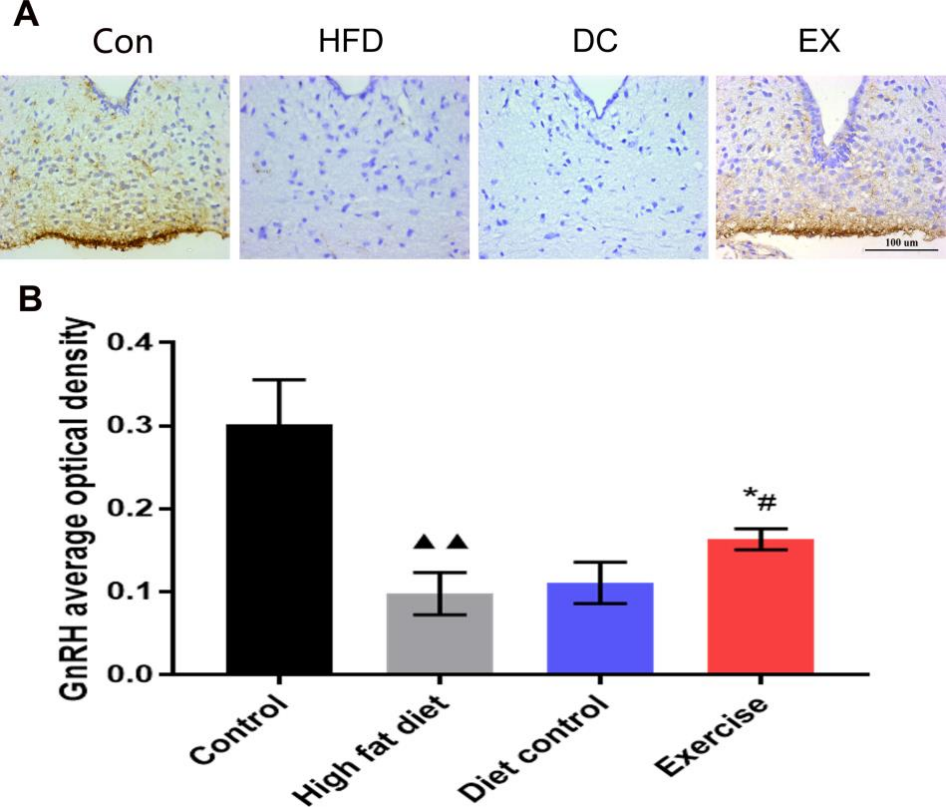
**The GnRH expression levels in the hypothalamic arcuate nucleus.** (**A**) GnRH positive expression in the brown area. The scale bar = 100 um. (**B**) The statistics of the GnRH average optical density value of each group. Image-Pro Plus 6.0 software was used to calculate the average optical density of GnRH. vs control group, ^**▲▲**^*p* < 0.01. vs HFD group, * p < 0.05, ** p < 0.01*.* vs DC group, ^*#*^*p* < 0.05, ^*##*^ *p* < 0.01*.*

It is estimated that hypothalamic inflammation is associated with the aging process. We assumed that the SIRT1-NF-κB pathway could affect the GnRH secretion in the hypothalamus and improve the physiological conditions during aging. Thus, the expression of GnRH in the hypothalamic arcuate nucleus was detected (Figure 6A). The average optical density value of GnRH in the HFD group decreased significantly compared with the control group (0.10 ± 0.03 vs. 0.30 ± 0.05, *p* < 0.01) (Figure 6B). The average optical density value of GnRH in the DC group increased slightly compared with the HFD group (Figure 6B). The levels of GnRH expression in the EX group was higher than the DC group and HFD group (0.16 ± 0.01 vs. 0.11 ± 0.02, *p* < 0.05. 0.16 ± 0.01 vs.0.10 ± 0.03, *p* < 0.05) (Figure 6B).

### Object recognition test (ORT) for learning and memory capabilities

**Figure 7:**
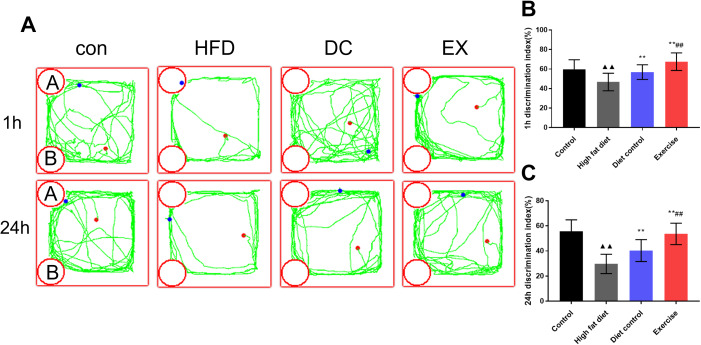
**ORT was used to detect the learning and memory ability of mice.** (**A**) Experimental trajectory map of mice exploring “A” and “B” objects in ORT device. (**B**) Comparison of 1 h identification index of mice in each group. (**C**) Comparison of 24 h identification index of mice in each group. vs control group, ^**▲▲**^*p* < 0.01. vs HFD group, ** *p* < 0.01. vs DC group, ^##^ *p* < 0.01.

Since the hypothalamic inflammation and GnRH levels may exert an effect on cognition, ORT was used to detect the learning and memory capabilities. The trajectory chart of mice during ORT was showed in the Figure 7A. The HFD group had the lower discrimination index (DI 1 h) than the control group (*p* < 0.01), 1 h DI of the DC and EX group increased gradually (*p* < 0.01), the EX group had higher 1 h DI than the DC group (*p* < 0.01) (Figure 7B). The similar results were observed in the ORT 24h test (Figure 7C). The results show that swimming and diet control can significantly improve the learning and memory ability of ApoE-/- mice that is associated with the inhibition of hypothalamic inflammation and promotion of GnRH release.

## DISCUSSION

The hypothalamus is the crucial center for the integration of peripheral signals with brain physiological functions [1, 16]. Accumulating evidence suggest that hypothalamus dysfunction is linked to aging metabolism, especially with early manifestations of Alzheimer's disease (AD) [17]. Do K et al. reported that metabolic abnormalities early occurred in the AD mice, which were tied to the over-expression of inflammation-related genes in the hypothalamus [18]. Diet and exercise are the crucial factors in aging and are closely interrelated to the inflammation of the hypothalamus [1, 3, 5]. The long-term high-fat diet can promote inflammation of the hypothalamic arcuate nucleus and induce systemic inflammation [19]. It is demonstrated that the activation of astrocytes and microglia could participate in the hypothalamic inflammation and exert an important role in systemic aging [9]. Astrocytes and microglia interact synergistically and are involved in neuro -inflammation [20]. A high-fat diet and sedentary lifestyle can activate microglia, influence hypothalamic inflammation and aging process, causing cognitive decline [21, 22]. Our experiments showed that a high-fat diet could increase peripheral inflammation and hypothalamic inflammation in ApoE -/- mice, activate glial cells and inhibit GnRH secretion, and lead to poor learning and memory. Swimming exercise and diet control can alleviate systemic inflammation and metabolism, improve dysfunction of the hypothalamus, promote GnRH secretion, and ameliorate cognition. However, the mechanism of swimming and diet improving hypothalamic inflammation and aging has not been elucidated.

SIRT1 is a Nicotinamide Adenine Dinucleotide (NAD) dependent deacetylase and known as a longevity gene. It has been linked to the prolongation of life span in many organisms and participation in neurodegenerative diseases [23]. Kumar et al. reported that the serum concentrations of SIRT1 steadily declined with age and dementia progression, indicating that serum SIRT1 may be a biomarker for the cognitive disease [24]. There were a low serum concentrations and gene expression of SIRT1 in overweight subjects, and calorie restriction could significantly increase serum SIRT1 concentrations [25]. Elevated SIRT1 activity can prevent endothelial cell from death and delay endothelial cell senescence [26]. SIRT1 activated the forkhead box O, PPARγ and Liver X receptor (LXR), regulated lipids metabolism and inhibited cholesterol deposit [11, 27]. It is estimated that the PPARγ-LXR-ATP binding cassette transporter A1(ABCA1) pathway is involved in promoting HDL synthesis and cholesterol efflux [28]. Several studies have shown that SIRT1 can activate PPARγ, SREBP-1c, LXR and ABCA1, and exert a key role in lipid metabolism [29–31]. It has been proved that exercise and calorie restriction could raise SIRT1 expression [11, 32, 33]. Our study found that the HFD group had lower level of SIRT1 expression, the peripheral level of SIRT1 expression was increased gradually in DC and EX group. Both DC and EX groups reduced the levels of TC, TG, LDL and glucose, and raised the levels of HDL gradually compared with HFD group. The results showed that swimming exercise plus diet control was more effective than diet control in improving peripheral SIRT1 expression, decreasing TG levels and increasing HDL levels. It indicates that swimming exercise plus diet control exerts an important role in lipid metabolism through SIRT1-LXR-ABCA1 pathways and promotes reverse cholesterol transport, elevates HDL generation and induces TG degradation [27–31]. SIRT1 exerts beneficial roles in regulating hepatic lipid metabolism through reverse cholesterol transport, relieving inflammation and lipids deposit in the liver [34]. Our results showed that the swimming and diet raised the SIRT1 expression and protected against the progression of lipids deposit in the liver. The higher SIRT1 expression means younger physiological conditions. Increased Sirt1 expression in the hypothalamus prevents neuromuscular junctions in skeletal muscle from age-related changes [35]. In the present study, we showed that a high-fat diet induced inflammatory cell infiltration into muscle, swimming exercise and diet control could relieve the inflammation of skeletal muscle (gastrocnemius).

Swimming exercise relieves the peripheral and central inflammation, but the mechanism has not been investigated extensively to date. It is reported that SIRT1 can inhibit nuclear factor kappa B (NF-κB) signaling pathway and suppress the systemic inflammation [11, 12, 31]. The NF-κB is composed of two subunits, P50 and P65, and is activated by various inflammatory cytokines. Activated NF-κB gets into the nucleus and promotes the transcription and translation of downstream factors like TNFα, IL-1β and apoptosis-related genes [32]. SIRT1 repressed the matrix metallo -proteinases (MMPs) expression by directly regulating the NF-κB signaling pathway or LXR-NF-κB pathway [31, 36]. Therefore, the results of our experiment showed that the EX group had the highest levels of SIRT1 expression and lowest levels of inflammatory cytokines (IL-1β, TNFα, MMP-2 and MMP-9) among groups. Our results further confirmed that swimming exercise plus diet control elevated SIRT1 expression and repressed the TNFα and MMP-9 expression more significantly than diet control. It suggests that swimming exercise plus diet control suppress peripheral inflammation that is linked to activation of SIRT1-LXR expression and inhibition of NF-κB activation.

Zhang et al. reported that NF-κB pathway in the hypothalamus was associated with systemic aging [4]. The overexpression of TNF-α and NF-κB were found in the activation of microglia of hypothalamus, which was involved in the aging process [4, 21, 22]. SIRT1 deacetylated the NF-κB subunit RelA/p65, resulted in low-expression of NF-κB, prevented the occurrence of inflammation [13]. Moderate training activated SIRT1 and inhibited NF-κB signal transmission [37]. SIRT1 overexpression and SIRT1 agonists dramatically inhibited the NF-κB pathway and have strong neuroprotective effects [38]. SIRT1 agonists resveratrol was recognized as anti-aging compounds, ameliorating aging-related progressive disease [39]. Activation of microglia and inflammation was suppressed by SIRT1 agonists [40]. Until now, the SIRT1-related hypothalamic molecular mechanism of swimming has not been investigated. In our study, high-fat diet repressed the expression of SIRT1 in the hypothalamus of ApoE-/- mice. The EX group had the highest levels of SIRT1 expression and lowest levels of NF-κB p65 expression in the hypothalamus. Arcuate nucleus of the hypothalamus which lacks of an integrated blood-brain barrier is easily disturbed by excess circulating free fatty acids and cytokines, causing inflammation and microglia activation of the hypothalamus [41]. Microglial activation induced interleukin-1 and TNF-α expression, then activated A1-reactive astrocytes and released neurotoxins and cytokines to promote apoptosis of neurons and oligodendrocytes [18, 20, 42]. Nogueira et al. showed that swimming could protect the synapses and reduce astrogliosis in the hypothalamus of high-fat diet animals [43]. We confirmed that the over-expression of TNF-α, IL-1β, MMP-2 and MMP-9 induced by high-fat diet in the hypothalamus could be suppressed by swimming exercise and diet control. High-fat diet initiated the activation of microglia and astrocytes in the hypothalamus of ApoE-/- mice. Swimming exercise and diet control mitigated the glial cells activation. These data indicates that swimming plus diet is more effective than diet control in suppressing inflammation and glial cells activation, which is associated with SIRT1-NF-κB pathway. NF-κB pathway in astrocyte is involved in the hypothalamic inflammation [44]. Hypothalamic astrocyte inflammation is linked to weight gain through activating of NF-κB signaling [45]. Our results showed that ApoE-/- mice gained body weight dramatically after high-fat diet feeding and swimming exercise plus diet could restrict more body weight gain than diet control, which may be related to hypothalamic astrocyte inflammation via NF-κB pathway. However, the phenotypic transformation of microglia and astrocytes in the hypothalamus has not been detected. The mechanism of glial cells in different polarization states deserves further research.

The SIRT1-NF-κB signaling pathway usually takes part in neurodegenerative and inflammatory diseases [46]. Inflammation is an important risk factor for age-related diseases [5]. A series of evidence suggest that aging is susceptible to dysfunction of the hypothalamus. Age-related inflammatory reaction can initiate activation of microglia [7, 8, 42]. The expression of SIRT1 in astrocytes contributes to reproductive regulation, which affects the GnRH secretion and action [47]. It has been demonstrated that inhibition of NF-κB in the hypothalamus increases the release of GnRH, extends the lifespan of mice [4]. Elevated GnRH levels in mice partially altered aging process and repressed hypothalamic inflammation [48]. GnRH improved various of aging effects and functional outcome measures, e.g. body weight, liver, muscle, skin, bone [48]. Our study found GnRH expression was down-regulated by high-fat diet in the hypothalamus. Swimming exercise plus diet control raised the expression of GnRH in the hypothalamus in high-fat diet ApoE-/- mice. Swimming and diet reduced the levels of lipids and glucose, reversed body weight gain and improved the morphology of liver and muscle in the high-fat diet ApoE-/- mice. It suggests that swimming and diet induce the secretion of GnRH in the hypothalamus against the aging related physiological changes, which is closely associated with the expression of SIRT1-NF-κB-GnRH.

Since GnRH levels is involved in aging and related with learning and memory ability, we detected the learning and memory performance of the mice by object recognition test (ORT). ORT can examine different stages of learning and memory (i.e. acquisition, merger or recall) to evaluate different types of memory, including spatial memory, short-term memory and long-term memory [49]. A decrease in the discrimination index (DI) suggests that the mice's spatial memory and learning ability are impaired [50]. This study found that the DI of 1 and 24 h was significantly decreased in the high-fat diet mice, indicating that learning and memory abilities were impaired. The DI in the diet control group and swimming exercise group increased gradually and the cognitive ability gradually improves. Swimming exercise plus diet control significantly enhanced the learning and memory abilities compared with diet control alone. High-fat diet induces the hypothalamic inflammation and inhibits the GnRH expression, altering hypothalamic outputs to hippocampus, amygdala, and reward-processing centers, which is disruption to cognitive function [41]. Swimming exercise and diet control improved the cognitive function in the high-fat diet mice, which may be linked to the expression of SIRT1-NF-κB-GnRH in the hypothalamus.

In summary, our experiments showed that a high-fat diet disturbed the lipids metabolism, induced systemic inflammation, activated glial cells and inhibited GnRH secretion in the hypothalamus of ApoE-/- mice. Swimming exercise and diet control reduced the peripheral and hypothalamic inflammation, suppressed glial cells activation, elevated GnRH secretion and improved the cognition, which was strongly related to the SIRT1-NF-κB expression in the hypothalamus. Taken together, our data identified that swimming and diet could improve the aging related physical conditions, and promote recovery of cognitive function, which is related to the expression of SIRT1-NF-κB-GnRH. It provides a new basic notion on diet and exercise for hypothalamus dysfunction during aging.

## MATERIALS AND METHODS

### Experimental animals

Adult Specific pathogen-free (SPF) ApoE-/- male mice (Nanjing Biomedical Research Institute of Nanjing University, Limited in Nanjing, China). The animal license number is SCXK (Su) 2015-001. All mice were placed in standard cages of SPF Laboratory of Experimental Animal Center of Fujian University of Traditional Chinese Medical (Fuzhou, China). Animals were free to get food and water in a 12/12 h light/dark cycle under a temperature control of 23 ± 2°C. The animal experiments were carried out by the approval of the Animal Management System and Use Committee of Fujian University of Traditional Chinese Medicine.

### Reagents

GnRH antibody (ab16216) and IL-1β antibody (ab9722) were purchased from Abcam (Cambridge, UK). NF-κB p65(D14E12, Catalog number:8242) was purchased from Cell Signaling Technology, Inc (Boston, USA). Iba1 antibody (Catalog number: 10904-1-AP), GFAP antibody (Catalog number:16825-1-AP), SIRT1 antibody (Catalog number:13161-1-AP), TNF-α antibody (Catalog number:17590-1-AP), GAPDH (Catalog number:60004-1-Ig) were purchased from Proteintech Group Inc (Wuhan, China). Total cholesterol assay kit (Catalog number:A111-1-1), Triglyceride assay kit (Catalog number:A110-1-1), Low-density lipoprotein cholesterol assay kit (Catalog number:A113-1-1), High-density lipoprotein cholesterol assay kit (Catalog number:A112-1-1) and Glucose assay kit (Catalog number:F006-1-1) were purchased from Nanjing Jiancheng Bioengineering Institute (Nanjing, China). ELISA Kits of mouse MMP-2, MMP-9, TNF-α and IL-1β (The catalog numbers: EK0460, EK0466, EK0527, EK0394) were purchased from BOSTER Biological Technology.co.ltd (Wuhan, China). High-fat diet (MD12015) was purchased from Jiangsu Medison Biomedical Co., Ltd (Yangzhou, China). Elisa kit of SIRT1 was purchased from Enzyme-free Biotechnology Co., Ltd (Jiangsu, China).

### Instrument

LEICA ordinary optical microscope (Leica Microsystems Co. Ltd., Germany); Tecan Infinite M200 Multifunctional Microplate Reader (Tecan Trading AG, Switzerland); Bio-Rad ChemiDoc XRS + ultra-high sensitivity chemiluminescence imaging system (Bio-Rad Laboratories, Inc., USA). Tecan Infinte M200 Microplate Reader (Switzerland). Object Recognition Test instrument (Shanghai Xinruan Information Technology Co., Ltd, China).

### Grouping of experimental animals

Thirty-six ApoE-/- mice (age 20weeks) were randomly divided into three groups: high-fat diet group (HFD), diet control group (DC), swimming group (EX). All of the mice were fed with high-fat diet (21% fat, 0.15% cholesterol) for 12 weeks and had free access to water. 12 wild-type mice of the same age and genetic background were randomly chosen as a control group and fed with normal diet.

### Intervention methods

After 12 weeks of high-fat or normal diet feeding, the HFD group continued to receive a high-fat diet. The DC group and the EX group were replaced with the normal diet. The EX group were subjected to swimming exercise at the same time. The swimming protocol is based on the research of Pellegrin’s [15]. The first week is the adaptation phase. The mice swam 10 minutes on the first day, then add 10 minutes each day. The mice swam for 50 minutes on the fifth day and again for 50 minutes on the sixth day. The formal training phase started from the second week. The mice swam for 50 minutes, once a day, 6 days a week for 7 weeks. The size of the swimming pool is 100cmx80cmx60cm with a 50cm water depth and the 30 ± 2°C water temperature. Mice were weighed weekly. The mice in the same cage swam in the same pool. During the swimming process, wooden rods were used to drive away floating mice that were not swimming. When the mice sink to the surface for more than 5 seconds and do not float up, training suspended. After each swimming training session, wipe off the water from the mice as soon as possible, dry the fur, clean the swimming pool, and return them to the original cage.

### Detection of serum lipids, SIRT1 and cytokines in serum

Animal tissue sample were collected after the intervention. Blood was removed from the eyeballs. The blood samples were placed in an ultra-low temperature high-speed centrifuge, centrifuged at 3000 rpm for 10 minutes at 4 °C, and the top serum was collected. Adding serum and standards to a 96-well plate that was incubated at 37 °C for 10 minutes according to the instructions in the total cholesterol (TC) assay kit (single-reagent GPO-PAP method).The absorbance value of each well was measured by a microplate reader at a wavelength of 510 nm. The concentration of total cholesterol in serum was calculated according to the formula. Each sample was repeated 3 times to calculate the average value. The same method was used to detect serum triglyceride (TG), low density lipoprotein (LDL), and high density lipoprotein (HDL) concentrations. Glucose was measured according to manufacturer protocol. Interleukin-1β (IL-1β), tumor necrosis factor-α (TNF-α), matrix metalloproteinase-2 (MMP-2), matrix metalloproteinase-9 (MMP-9) in serum was measured by the Elisa kits. Sirtuin (SIRT1) levels in serum was detected according to the instructions.

### Western blot (WB) analysis

An appropriate amount of lysate was added to the tissue. The protein supernatant was extracted after ultrasonic grinding and centrifugation. BCA method was used to detect the concentration of protein supernatant, and denatured in a metal bath at 100 °C. Configure 10%, 12% separation gel and 5% concentrated gel according to the instructions of the electrophoresis gel kit. After the sample was loaded, electrophoresis was performed, and a band corresponding to the molecular weight was cut according to the Marker position to transfer the membrane. Then, the specific site was blocked with BSA, and the membrane was incubated with primary antibodies GAPDH (1:10000), SIRT1 (1:800), NF-κB p65 (1:1000), TNFα (1:1000), and IL-1β (1:1000) overnight at 4 °C. ECL was developed after secondary antibody (1:5000) was shaken at room temperature for 1 h. The software calculates the band gray value to analyze the protein expression.

### Immunohistochemical staining

The brain was fixed by cardiac perfusion with 4% paraformaldehyde and removed for paraffin embedding. A 5 μm paraffin section of brain tissue was dewaxed. Antigen recovery was performed with sodium citrate repair solution. Then, according to the immunohistochemical assay, endogenous peroxide was added for 10 min and non-specific stain blocking agents were added. Primary antibodies Iba1, GFAP, and GnRH were added overnight. Biotin-labeled secondary antibodies were added. Then observe under the microscope for DAB staining to control the time, counterstain with hematoxylin, and seal with neutral resin after washing with water, observe under the microscope, and take pictures.

### HE staining for the morphological changes of liver and gastrocnemius

Paraffin embedding of the liver and gastrocnemius muscle was performed in the same way as the brain tissue. The tissue was cut into 5 μm sections. The cutting rack was immersed in hematoxylin staining solution. The remaining hematoxylin solution was rinsed under running water and immersed in eosin staining solution. The staining time of hematoxylin and eosin staining solution was checked under a microscope. After the slides are dried, neutral resin gasket. Randomly select the field of view under the light microscope and capture images.

### ORT detection learning and memory capabilities

Behavioral testing was conducted according to the manufacturer's protocol. In brief, three objects numbered “a” ,”A” and “B” were prepared. Object “a” is the same with “A”, different from “B”. The mice were placed in the test room and adapted to the environment before the test. Place the same objects “a” and “A” on the left and right sides. The mice were placed in the field with the back of the two objects. The test lasted ten minutes. The exploration trajectory and the contact with the two objects of mice were recorded. Recorded as “Ta” and “TA”. Next step, replace the object in the field with the “B” object, “TA” and “TB” were recorded. The observation of mice on the new object “B” was mainly observed and marked with the discrimination index, the discrimination index = TB / (TA + TB).

### Statistical analysis

The experimental data were statistically analyzed using IBM SPSS software version 20.0 (SPSS, Chicago, IL, USA). And the data were expressed using mean ± SD. The statistical analysis was performed by t-test and one-way analysis of variance, followed by the LSD test when necessary. *p*<0.05 was considered to indicate statistical significance.

## Supplementary Material

Supplementary Table 1

## References

[1] Schafer MJ, White TA, Evans G, Tonne JM, Verzosa GC, Stout MB, Mazula DL, Palmer AK, Baker DJ, Jensen MD, Torbenson MS, Miller JD, Ikeda Y, et al. Exercise prevents diet-induced cellular senescence in adipose tissue. Diabetes. 2016; 65:1606–15. 10.2337/db15-0291 26983960 PMC4878429

[2] Jais A, Brüning JC. Hypothalamic inflammation in obesity and metabolic disease. J Clin Invest. 2017; 127:24–32. 10.1172/JCI88878 28045396 PMC5199695

[3] Purkayastha S, Cai D. Neuroinflammatory basis of metabolic syndrome. Mol Metab. 2013; 2:356–63. 10.1016/j.molmet.2013.09.005 24327952 PMC3854982

[4] Zhang G, Li J, Purkayastha S, Tang Y, Zhang H, Yin Y, Li B, Liu G, Cai D. Hypothalamic programming of systemic ageing involving IKK-β, NF-κB and GnRH. Nature. 2013; 497:211–16. 10.1038/nature12143 23636330 PMC3756938

[5] Beavers KM, Brinkley TE, Nicklas BJ. Effect of exercise training on chronic inflammation. Clin Chim Acta. 2010; 411:785–93. 10.1016/j.cca.2010.02.069 20188719 PMC3629815

[6] Ferrer MD, Capó X, Martorell M, Busquets-Cortés C, Bouzas C, Carreres S, Mateos D, Sureda A, Tur JA, Pons A. Regular practice of moderate physical activity by older adults ameliorates their anti-inflammatory status. Nutrients. 2018; 10:1780. 10.3390/nu10111780 30453505 PMC6266855

[7] Klein C, Jonas W, Wiedmer P, Schreyer S, Akyüz L, Spranger J, Hellweg R, Steiner B. High-fat diet and physical exercise differentially modulate adult neurogenesis in the mouse hypothalamus. Neuroscience. 2019; 400:146–56. 10.1016/j.neuroscience.2018.12.037 30599265

[8] Silva VR, Katashima CK, Lenhare L, Silva CG, Morari J, Camargo RL, Velloso LA, Saad MA, da Silva AS, Pauli JR, Ropelle ER. Chronic exercise reduces hypothalamic transforming growth factor-β1 in middle-aged obese mice. Aging (Albany NY). 2017; 9:1926–40. 10.18632/aging.101281 28854149 PMC5611986

[9] Leite MR, Cechella JL, Pinton S, Nogueira CW, Zeni G. A diphenyl diselenide-supplemented diet and swimming exercise promote neuroprotection, reduced cell apoptosis and glial cell activation in the hypothalamus of old rats. Exp Gerontol. 2016; 82:1–7. 10.1016/j.exger.2016.05.006 27215802

[10] Marinho R, Munõz VR, Pauli LS, Ropelle EC, de Moura LP, Moraes JC, Moura-Assis A, Cintra DE, da Silva AS, Ropelle ER, Pauli JR. Endurance training prevents inflammation and apoptosis in hypothalamic neurons of obese mice. J Cell Physiol. 2018; 234:880–90. 10.1002/jcp.26909 30078194

[11] Fujita Y, Yamashita T. Sirtuins in neuroendocrine regulation and neurological diseases. Front Neurosci. 2018; 12:778. 10.3389/fnins.2018.00778 30416425 PMC6213750

[12] Chen WK, Tsai YL, Shibu MA, Shen CY, Chang-Lee SN, Chen RJ, Yao CH, Ban B, Kuo WW, Huang CY. Exercise training augments Sirt1-signaling and attenuates cardiac inflammation in d-galactose induced-aging rats. Aging (Albany NY). 2018; 10:4166–74. 10.18632/aging.101714 30582744 PMC6326662

[13] Hwang JW, Yao H, Caito S, Sundar IK, Rahman I. Redox regulation of SIRT1 in inflammation and cellular senescence. Free Radic Biol Med. 2013; 61:95–110. 10.1016/j.freeradbiomed.2013.03.015 23542362 PMC3762912

[14] Fukao K, Shimada K, Naito H, Sumiyoshi K, Inoue N, Iesaki T, Kume A, Kiyanagi T, Hiki M, Hirose K, Matsumori R, Ohsaka H, Takahashi Y, et al. Voluntary exercise ameliorates the progression of atherosclerotic lesion formation via anti-inflammatory effects in apolipoprotein e-deficient mice. J Atheroscler Thromb. 2010; 17:1226–36. 10.5551/jat.4788 20808053

[15] Pellegrin M, Berthelot A, Houdayer C, Gaume V, Deckert V, Laurant P. New insights into the vascular mechanisms underlying the beneficial effect of swimming training on the endothelial vasodilator function in apolipoprotein e-deficient mice. Atherosclerosis. 2007; 190:35–42. 10.1016/j.atherosclerosis.2006.02.001 16529753

[16] Markakis EA. Development of the neuroendocrine hypothalamus. Front Neuroendocrinol. 2002; 23:257–91. 10.1016/s0091-3022(02)00003-1 12127306 PMC3242412

[17] Hiller AJ, Ishii M. Disorders of body weight, sleep and circadian rhythm as manifestations of hypothalamic dysfunction in alzheimer’s disease. Front Cell Neurosci. 2018; 12:471. 10.3389/fncel.2018.00471 30568576 PMC6289975

[18] Do K, Laing BT, Landry T, Bunner W, Mersaud N, Matsubara T, Li P, Yuan Y, Lu Q, Huang H. The effects of exercise on hypothalamic neurodegeneration of alzheimer’s disease mouse model. PLoS One. 2018; 13:e0190205. 10.1371/journal.pone.0190205 29293568 PMC5749759

[19] Thaler JP, Yi CX, Schur EA, Guyenet SJ, Hwang BH, Dietrich MO, Zhao X, Sarruf DA, Izgur V, Maravilla KR, Nguyen HT, Fischer JD, Matsen ME, et al. Obesity is associated with hypothalamic injury in rodents and humans. J Clin Invest. 2012; 122:153–62. 10.1172/JCI59660 22201683 PMC3248304

[20] Liddelow SA, Barres BA. Reactive astrocytes: production, function, and therapeutic potential. Immunity. 2017; 46:957–67. 10.1016/j.immuni.2017.06.006 28636962

[21] Yin Z, Raj DD, Schaafsma W, van der Heijden RA, Kooistra SM, Reijne AC, Zhang X, Moser J, Brouwer N, Heeringa P, Yi CX, van Dijk G, Laman JD, et al. Low-fat diet with caloric restriction reduces white matter microglia activation during aging. Front Mol Neurosci. 2018; 11:65. 10.3389/fnmol.2018.00065 29593493 PMC5857900

[22] Tapia-González S, García-Segura LM, Tena-Sempere M, Frago LM, Castellano JM, Fuente-Martín E, García-Cáceres C, Argente J, Chowen JA. Activation of microglia in specific hypothalamic nuclei and the cerebellum of adult rats exposed to neonatal overnutrition. J Neuroendocrinol. 2011; 23:365–70. 10.1111/j.1365-2826.2011.02113.x 21314736

[23] Chong ZZ, Shang YC, Wang S, Maiese K. SIRT1: new avenues of discovery for disorders of oxidative stress. Expert Opin Ther Targets. 2012; 16:167–78. 10.1517/14728222.2012.648926 22233091 PMC3279588

[24] Dong SF, Zhang SW, Sun JN. Silent mating-type information regulator 2 homolog 1(SIRT1) in Alzheimer's disease: an update on potential mechanisms. Chinese Pharmacol Bulletin. 2016; 32:1041–44. 10.3969/j.issn.1001-1978.2016.08.002

[25] Mansur AP, Roggerio A, Goes MF, Avakian SD, Leal DP, Maranhão RC, Strunz CM. Serum concentrations and gene expression of sirtuin 1 in healthy and slightly overweight subjects after caloric restriction or resveratrol supplementation: a randomized trial. Int J Cardiol. 2017; 227:788–94. 10.1016/j.ijcard.2016.10.058 28029409

[26] Hou J, Wang S, Shang YC, Chong ZZ, Maiese K. Erythropoietin employs cell longevity pathways of SIRT1 to foster endothelial vascular integrity during oxidant stress. Curr Neurovasc Res. 2011; 8:220–35. 10.2174/156720211796558069 21722091 PMC3149772

[27] Li Y, Wu S. Epigallocatechin gallate suppresses hepatic cholesterol synthesis by targeting SREBP-2 through SIRT1/FOXO1 signaling pathway. Mol Cell Biochem. 2018; 448:175–85. 10.1007/s11010-018-3324-x 29446047

[28] Chawla A, Boisvert WA, Lee CH, Laffitte BA, Barak Y, Joseph SB, Liao D, Nagy L, Edwards PA, Curtiss LK, Evans RM, Tontonoz P. A PPAR gamma-LXR-ABCA1 pathway in macrophages is involved in cholesterol efflux and atherogenesis. Mol Cell. 2001; 7:161–71. 10.1016/s1097-2765(01)00164-2 11172721

[29] Hammer SS, Beli E, Kady N, Wang Q, Wood K, Lydic TA, Malek G, Saban DR, Wang XX, Hazra S, Levi M, Busik JV, Grant MB. The mechanism of diabetic retinopathy pathogenesis unifying key lipid regulators, sirtuin 1 and liver X receptor. EBioMedicine. 2017; 22:181–90. 10.1016/j.ebiom.2017.07.008 28774737 PMC5552206

[30] Defour A, Dessalle K, Castro Perez A, Poyot T, Castells J, Gallot YS, Durand C, Euthine V, Gu Y, Béchet D, Peinnequin A, Lefai E, Freyssenet D. Sirtuin 1 regulates SREBP-1c expression in a LXR-dependent manner in skeletal muscle. PLoS One. 2012; 7:e43490. 10.1371/journal.pone.0043490 22984430 PMC3439460

[31] Zeng HT, Fu YC, Yu W, Lin JM, Zhou L, Liu L, Wang W. SIRT1 prevents atherosclerosis via liver-X-receptor and NF-κB signaling in a U937 cell model. Mol Med Rep. 2013; 8:23–28. 10.3892/mmr.2013.1460 23652462

[32] Hajighasem A, Farzanegi P, Mazaheri Z, Naghizadeh M, Salehi G. Effects of resveratrol, exercises and their combination on Farnesoid X receptor, liver X receptor and sirtuin 1 gene expression and apoptosis in the liver of elderly rats with nonalcoholic fatty liver. PeerJ. 2018; 6:e5522. 10.7717/peerj.5522 30221089 PMC6136396

[33] Igarashi M, Guarente L. mTORC1 and SIRT1 cooperate to foster expansion of gut adult stem cells during calorie restriction. Cell. 2016; 166:436–50. 10.1016/j.cell.2016.05.044 27345368

[34] Ding RB, Bao J, Deng CX. Emerging roles of SIRT1 in fatty liver diseases. Int J Biol Sci. 2017; 13:852–67. 10.7150/ijbs.19370 28808418 PMC5555103

[35] Snyder-Warwick AK, Satoh A, Santosa KB, Imai SI, Jablonka-Shariff A. Hypothalamic Sirt1 protects terminal schwann cells and neuromuscular junctions from age-related morphological changes. Aging Cell. 2018; 17:e12776. 10.1111/acel.12776 29851253 PMC6052483

[36] Liu P, Wilson MJ. miR-520c and miR-373 upregulate MMP9 expression by targeting mTOR and SIRT1, and activate the ras/raf/MEK/erk signaling pathway and NF-κB factor in human fibrosarcoma cells. J Cell Physiol. 2012; 227:867–76. 10.1002/jcp.22993 21898400 PMC3225649

[37] Liu HW, Chang SJ. Moderate exercise suppresses NF-κB signaling and activates the SIRT1-AMPK-PGC1α axis to attenuate muscle loss in diabetic db/db mice. Front Physiol. 2018; 9:636. 10.3389/fphys.2018.00636 29896118 PMC5987703

[38] Chen J, Zhou Y, Mueller-Steiner S, Chen LF, Kwon H, Yi S, Mucke L, Gan L. SIRT1 protects against microglia-dependent amyloid-beta toxicity through inhibiting NF-kappaB signaling. J Biol Chem. 2005; 280:40364–74. 10.1074/jbc.M509329200 16183991

[39] Sarubbo F, Esteban S, Miralles A, Moranta D. Effects of resveratrol and other polyphenols on Sirt1: relevance to brain function during aging. Curr Neuropharmacol. 2018; 16:126–36. 10.2174/1570159X15666170703113212 28676015 PMC5883375

[40] Zhang F, Liu J, Shi JS. Anti-inflammatory activities of resveratrol in the brain: role of resveratrol in microglial activation. Eur J Pharmacol. 2010; 636:1–7. 10.1016/j.ejphar.2010.03.043 20361959

[41] Miller AA, Spencer SJ. Obesity and neuroinflammation: a pathway to cognitive impairment. Brain Behav Immun. 2014; 42:10–21. 10.1016/j.bbi.2014.04.001 24727365

[42] Tang Y, Le W. Differential roles of M1 and M2 microglia in neurodegenerative diseases. Mol Neurobiol. 2016; 53:1181–94. 10.1007/s12035-014-9070-5 25598354

[43] Nogueira PA, Pereira MP, Soares JJ, de Assis Silva Gomes J, Ribeiro DL, Razolli DS, Velloso LA, Neto MB, Zanon RG. Swimming reduces fatty acids-associated hypothalamic damage in mice. J Chem Neuroanat. 2020; 103:101713. 10.1016/j.jchemneu.2019.101713 31726089

[44] Buckman LB, Thompson MM, Lippert RN, Blackwell TS, Yull FE, Ellacott KL. Evidence for a novel functional role of astrocytes in the acute homeostatic response to high-fat diet intake in mice. Mol Metab. 2014; 4:58–63. 10.1016/j.molmet.2014.10.001 25685690 PMC4314532

[45] Douglass JD, Dorfman MD, Fasnacht R, Shaffer LD, Thaler JP. Astrocyte IKKβ/NF-κB signaling is required for diet-induced obesity and hypothalamic inflammation. Mol Metab. 2017; 6:366–73. 10.1016/j.molmet.2017.01.010 28377875 PMC5369266

[46] Kaewmool C, Kongtawelert P, Phitak T, Pothacharoen P, Udomruk S. Protocatechuic acid inhibits inflammatory responses in LPS-activated BV2 microglia via regulating SIRT1/NF-κB pathway contributed to the suppression of microglial activation-induced PC12 cell apoptosis. J Neuroimmunol. 2020; 341:577164. 10.1016/j.jneuroim.2020.577164 32007785

[47] Choi I, Rickert E, Fernandez M, Webster NJ. SIRT1 in astrocytes regulates glucose metabolism and reproductive function. Endocrinology. 2019; 160:1547–60. 10.1210/en.2019-00223 31127273 PMC6542483

[48] Tang Y, Cai D. Hypothalamic inflammation and GnRH in aging development. Cell Cycle. 2013; 12:2711–12. 10.4161/cc.26054 23966154 PMC3899179

[49] Antunes M, Biala G. The novel object recognition memory: neurobiology, test procedure, and its modifications. Cogn Process. 2012; 13:93–110. 10.1007/s10339-011-0430-z 22160349 PMC3332351

[50] Bevins RA, Besheer J. Object recognition in rats and mice: a one-trial non-matching-to-sample learning task to study ‘Recognition memory’. Nat Protoc. 2006; 1:1306–11. 10.1038/nprot.2006.205 17406415

