# Supplementary material for: The effect of swimming exercise and diet on the hypothalamic inflammation of ApoE-/- mice based on SIRT1-NF-κB-GnRH expression
Source: Aging (Albany NY). 2020 Jun 9;12(11):11085–99. doi: 10.18632/aging.103323 (PMC7346084; doi:10.18632/aging.103323)
Supplement: Supplementary Table 1 [file aging-12-103323-s001..pdf]

SUPPLEMENTARY TABLE

Supplementary Table 1. Comparison of serum lipid levels (mmol / L; means ± SD).

Group	n	TC	TG	LDL	HDL
ApoE-/- normal diet	6	34.86±4.08	1.02±0.07	9.17±0.81	2.52±0.57
ApoE-/- High fat diet	6	59.87±14.66**	1.80±0.44**	19.94±1.89**	1.51±0.38**
P		<0.01	<0.01	<0.01	<0.01

12 weeks old ApoE-/-mice fed with high-fat diet for 6 weeks. Compared with ApoE-/- normal diet group, ** $p < 0.01$.
(Chinese Pharmacological Bulletin 2020 Apr;36(4)).
